# Intracellular glutathione availability, rather than vitamin C supplementation, determines endothelial cell resistance to water-soluble cigarette smoke toxicants

**DOI:** 10.3389/fphar.2026.1924584

**Published:** 2026-10-28

**Authors:** Gloria Oñate, Perla Salgado, Paula A. Guzmán, Alejandra Muñoz, Catalina Alarcon-Navarro, Solange P. Arriagada, Gustavo Moraga-Cid, Carola Muñoz-Montesino, Marcell Gatica, Coralia I. Rivas

**Affiliations:** 1 Departamento de Fisiopatología, Facultad de Ciencias Biológicas, Universidad de Concepción, Concepción, Chile; 2 Departamento de Morfología y Función, Facultad de Salud y Ciencias Sociales, Universidad de Las Américas, Concepción, Chile; 3 Departamento de Bioquímica y Biología Molecular, Facultad de Ciencias Biológicas, Universidad de Concepción, Concepción, Chile; 4 Departamento de Fisiología, Facultad de Ciencias Biológicas, Universidad de Concepción, Concepción, Chile; 5 Departamento de Ciencias Biológicas, Facultad de Ciencias de la Vida, Universidad Andrés Bello, Talcahuano, Chile

**Keywords:** cigarette smoke, endothelial cells, glutathione, oxidative stress, vitamin C

## Abstract

Smoking involves chronic exposure to cigarette smoke toxicants, which is associated with the development of vascular disease characterized by endothelial dysfunction. We examined the role of glutathione and vitamin C in antioxidant defense in endothelial cells exposed to water-soluble cigarette smoke toxicants. Exposure to cigarette smoke-conditioned medium decreased cell viability in a concentration and time-dependent manner, with rapid glutathione depletion and recovery. Cells exposed to non-lethal concentrations recovered their glutathione levels in a manner related with enhanced cell survival, and disruption of glutathione metabolism with L-buthionine-(S,R)-sulfoximine (BSO) drastically decreased cellular resistance to cigarette smoke toxicity and increased cell death. These negative effects were augmented in cells treated with graded hydrogen peroxide concentrations following exposure to cigarette smoke-conditioned medium. Intracellular vitamin C did not provide sustained protection against cigarette smoke toxicity and did not enhance glutathione recovery following oxidative challenge. Moreover, exposure to cigarette smoke-conditioned medium failed to affect the transport of reduced (ascorbic acid) or oxidized (dehydroascorbic acid) vitamin C by endothelial cells, regardless of the status of intracellular glutathione or vitamin C. We conclude that glutathione and vitamin C possess overlapping but clearly distinct functions in the response of cells to oxidative challenge. Specifically, in endothelial cells exposed to soluble cigarette smoke toxicants, glutathione was the major determinant against cigarette smoke toxicity.

## Introduction

Smoking is one of the leading causes of mortality and morbidity in the modern world, with more than 2 billion smokers worldwide (Collaborators, 2020; Collaborators, 2021; Jha, 2009). Smoking involves long-term exposure to cigarette smoke toxicants (Adam et al., 2009), which is associated with systemic events that include the development of chronic vascular disease, a complex syndrome that is characterized by endothelial dysfunction and altered response to oxidative stress (Endemann and Schiffrin, 2004). Current evidence further indicates that endothelial dysfunction induced by smoking is strongly linked to reduced nitric oxide bioavailability, increased oxidative stress, and activation of pro-inflammatory and pro-thrombotic pathways (Ishida et al., 2024; Ueda et al., 2023).

Vitamin C protects endothelial cells from oxidative stress by neutralizing the effects of oxidative species and by decreasing blood cell-endothelial cell interactions. Glutathione modulates the redox properties of vitamin C in endothelial cells (Montecinos et al., 2007). Long-term cigarette smoke exposure may lead to the development of systemic, extrapulmonary effects that include an imbalance of the normal oxidant-antioxidant homeostasis, increased products of lipid peroxidation, low-grade systemic inflammatory response and depleted plasma levels of antioxidants (Ashor et al., 2014; Astori et al., 2022; Bruno et al., 2005; Dietrich et al., 2003; Karademirci et al., 2018; Lykkesfeldt and Tveden-Nyborg, 2019; Panda et al., 2000; Preston et al., 2003; Strauss, 2001; Wilson et al., 2011). More recent studies have emphasized that cigarette smoke contains high concentrations of reactive oxygen species and electrophilic compounds that sustain a chronic state of oxidative stress and inflammation, thereby amplifying vascular injury (Seo et al., 2023).

The lower plasma vitamin C levels in smokers can be reversed by moderate supplementation with the vitamin that improves endothelial function (Ashor et al., 2014). However, evidence suggests that vitamin C supplementation alone may be insufficient to fully restore redox balance in smokers due to persistent oxidative burden (Raitakari et al., 2000). Many studies indicate a role for glutathione in the protection against cigarette smoke (Baglole et al., 2008; Conklin et al., 2009; Dalle-Donne et al., 2020; Horiyama et al., 2016; Luchese et al., 2009; Muscat et al., 2004; Shin et al., 2020; van der Toorn et al., 2007) and show that cigarette smoke exposure can deplete the available intracellular glutathione pool by irreversibly generating glutathione-aldehyde derivatives (Dalle-Donne et al., 2020; Horiyama et al., 2016; van der Toorn et al., 2007). This depletion has been associated with impaired detoxification capacity and increased susceptibility to oxidative damage in vascular cells (Conklin et al., 2009).

Vitamin C and glutathione differ in their cellular availability, recycling, and detoxification mechanisms. Humans are unable to synthesize vitamin C and therefore must obtain it from dietary sources and cellular uptake through specific membrane transport systems (Corti et al., 2010; Nishikimi et al., 1994; Rivas et al., 2008), whereas glutathione is synthesized intracellularly (Lu, 2013). Ascorbic acid undergoes reversible oxidation and can be recycled intracellularly (Linster and Van Schaftingen, 2007; Nualart et al., 2003), while glutathione participates not only in redox reactions but also in the detoxification of electrophilic xenobiotics (Blair, 2006; Dourado et al., 2008; Hayes et al., 2005). In this latter process, GSH can form covalent adducts that may be exported from cells, thereby decreasing the available intracellular glutathione pool (Dourado et al., 2008; van der Toorn et al., 2007).

The functional interaction between glutathione and vitamin C has also been demonstrated experimentally. Disruption of glutathione metabolism *in vivo* in rats and in guinea pigs by treatment with buthionine-(SR)-sulfoximine (BSO), a potent and specific inhibitor of glutathione synthesis inhibitor (Hamilton et al., 2007), revealed that the dysfunction and mortality associated with glutathione deficiency can be ameliorated by supplementation with vitamin C (Martensson and Meister, 1991). Conversely, glutathione ester supplementation delayed the effects of a vitamin C-free diet in newborn rats and guinea pigs unable to synthesize vitamin C (Martensson et al., 1993). In endothelial cells subjected to oxidative challenge in which glutathione may exceed vitamin C by more than one order of magnitude on a molar basis, full antioxidant protection requires the simultaneous presence of intracellular and extracellular vitamin C at concentrations that are normally observed *in vivo* (Montecinos et al., 2007).

Despite the well-established functional interaction between glutathione and vitamin C (Mardones et al., 2012; Montecinos et al., 2007; Tram et al., 2021), their relative contribution to endothelial cell protection during exposure to soluble cigarette smoke remain poorly understood. In particular, it remains unclear whether vitamin C can compensate sufficient antioxidant protection for decreased intracellular glutathione availability during sustained oxidative stress challenge (Guaiquil et al., 2001; Shang et al., 2003).

In this study, we characterized the relative contribution of glutathione and vitamin C in the antioxidant defense of human umbilical vein endothelial cells (HUVECs) exposed to cigarette smoke-conditioned culture medium. We examined the relationship between intracellular glutathione availability, vitamin C status, and endothelial cell survival during acute and prolonged exposure to soluble cigarette smoke toxicants.

## Experimental procedures

### Conditioned medium

Cigarette smoke-conditioned medium (CSM) was prepared by bubbling the smoke of commercially available filter cigarettes (Belmont cigarette, British American Cigarette Chile; nicotine: 0.6 mg; tar: 7 mg; 8 cm long, 3 cm filter) into cell culture medium prewarmed at 37 °C. The cigarette was inserted into a plastic tube (10-cm length) that was attached to a glass tube submerged in a flask containing 20 mL of cell culture medium. We used an aluminium foil-covered kitasato flask and negative pressure from a peristaltic pump calibrated to ensure that each cigarette was “smoked” in 5 min. Because no standardized method is available for expressing the concentration of water-soluble cigarette smoke components, concentrations were expressed as cigarette equivalents per milliliter of culture medium. In a standard procedure, the smoke of 4 cigarettes was bubbled into 20 mL of culture medium to collect the water-soluble components of the cigarette smoke, yielding a stock concentration of 0.2 cigarette/mL. The pH of the conditioned medium was adjusted to 7.5, filtered and frozen in aliquots that were thawed right before each experiment and used only once (Jia et al., 2024; Raveendran et al., 2005; Wang et al., 2025). For the experiments, the stock solution was diluted to a final concentration of 0.002 cigarette/mL, that we defined as one cigarette smoke equivalent (1 Eq), according to Raveendran et al. (2005). For longer-term exposure (7 days), the culture medium was replaced daily with fresh conditioned medium.

### Cell culture

Primary human umbilical vein endothelial cells (HUVECs), originally isolated from human umbilical cords, were obtained from pre-existing fully anonymized cell cultures. HUVECs were cultured in HE-SFM medium (Human Endothelial Serum-Free Medium, Gibco), supplemented with 20% fetal calf serum, L-glutamine, penicillin, streptomycin, and Fungizone, and were used at passages 3–4, as previously described (Montecinos et al., 2007).

### Glutathione depletion

To induce glutathione depletion, cells were incubated with L-buthionine-(S,R)-sulfoximine (BSO) for 24 h, followed by a 1-h incubation with diethyl maleate (DEM). The glutathione-depleting agents were applied only during this initial 24 + 1-h treatment period and were not maintained throughout the 7-day experiment (24BSO). In certain experiments, cells were treated with BSO daily, during the 7 days experiment (BSO7). After completion of the depletion protocol, cells were maintained under the corresponding experimental conditions and monitored for up to 7 days. This procedure reduced intracellular glutathione levels from approximately 2–3 mM in control cells to less than 50 µM in treated cells without affecting cell viability (Montecinos et al., 2007). Total cellular glutathione was quantified using the enzymatic recycling assay with 5, 5′-dithiobis-(2-nitrobenzoic acid) (DTNB), as previously described (Guaiquil et al., 1997; Montecinos et al., 2007).

### Quantification of intracellular glutathione

Intracellular acid-soluble glutathione, comprising soluble GSH plus GSSG, defined as the sum of reduced glutathione (GSH) and glutathione disulfide (GSSG), was quantified using a modified enzymatic recycling assay based on the method previously described (Baker et al., 1990). The assay measures the rate of formation of 5-thio-2-nitrobenzoic acid (TNB), a chromogenic product that absorbs at 412 nm. In this reaction, GSH reduces 5, 5′-dithiobis-(2-nitrobenzoic acid) (DTNB), resulting in the formation of TNB and GSSG. The newly formed GSSG is subsequently reduced back to GSH by glutathione reductase in the presence of NADPH. The regenerated GSH reacts again with DTNB, producing a continuous enzymatic recycling process. Because DTNB, NADPH, and glutathione reductase are present in excess, the rate of TNB formation is directly proportional to the total amount of glutathione in the sample. Cells grown in 24-well culture plates were lysed in 30 µL of 0.4% Triton X-100. Cell lysates were collected and transferred to microcentrifuge tubes containing an equal volume of 5% sulfosalicylic acid to precipitate proteins and preserve glutathione. Samples were centrifuged at 10,000 rpm for 2 min at 4 °C, and the resulting supernatants were stored at −80 °C until analysis. Before measurement, samples were maintained at 4 °C. The assay was performed in a final reaction volume of 150 µL containing 5 µL of 5 mM DTNB, 117 µL of phosphate buffer composed of 70 mM sodium phosphate and 3.9 mM EDTA, pH 7.5, 5 µL of 6 mM NADPH, and 18 µL of sample supernatant or glutathione standard. The reaction was initiated by adding 5 µL of glutathione reductase at 20 U/mL. Absorbance at 412 nm was then recorded every 30 s for 10 min. DTNB, NADPH, samples, and standards were maintained at 4 °C before initiation of the reaction. Glutathione standards ranging from 5 to 100 µM were prepared in 2.5% sulfosalicylic acid, such that both standards and samples contained a final sulfosalicylic acid concentration of 0.3% in the 150-µL assay. The assay blank was prepared under the same conditions. A new calibration curve was generated for each experiment by plotting the rate of change in absorbance at 412 nm against the corresponding glutathione standard concentration. The rate of TNB formation was calculated from the slope of the linear portion of each absorbance-versus-time curve. Glutathione concentrations in experimental samples were determined by interpolation from the corresponding calibration curve. Results were expressed as intracellular acid-soluble glutathione (GSH + GSSG) concentration in millimolar units. Intracellular glutathione concentrations ≤ 0.1 mM, corresponding to approximately 3%–5% of the levels measured in untreated cells, were operationally defined as severe glutathione depletion.

### Vitamin C treatment

To obtain vitamin C-loaded cells, cells were seeded into 12-well plates and cultured in complete growth medium until they reached confluence (1–2 days) (Nualart et al., 2003). Twenty-four hours before the experiment, cells were supplemented with L-ascorbic acid 2-phosphate (AA2P) at a final concentration of 100 μM. AA2P was used because it is stable under cell culture conditions (Manesia et al., 2023). Following hydrolysis by membrane-bound alkaline phosphatase, AA2P releases ascorbic acid, which is subsequently transported into the intracellular compartment (Takamizawa et al., 2004). During long-term experiments, the culture medium was replenished every 24 h with fresh medium containing 100 µM AA2P to maintain intracellular vitamin C levels. Ascorbic acid transport was measured using ^14^C radiolabeled L-ascorbic acid. For dehydroascorbic acid uptake experiments, radiolabeled DHA was generated immediately before the assay by oxidation of L-[^14^C] ascorbic acid with ascorbate oxidase, as previously described (Montecinos et al., 2007).

### Cell exposure to cigarette smoke

For all experiments, HUVECs were initially cultured in 12-well plates using smoke-free culture medium. At the beginning of the treatment, the medium was replaced with cigarette smoke-conditioned medium (CSM). For short-term exposure experiments, cells were incubated with CSM containing different cigarette smoke equivalent concentrations (0.1–10 equivalents) for the indicated time periods. For long-term exposure (1 week), the culture medium was replaced every 24 h with fresh CSM containing 1 cigarette smoke equivalent. In other experiments, the cells were exposed for 1 h to cigarette smoke-conditioned medium followed by a 15 min treatment with different hydrogen peroxide concentrations.

### Cell exposure to hydrogen peroxide

HUVECs were seeded into 12-well plates and cultured in complete growth medium containing fetal bovine serum until a confluent monolayer was established (1–2 days). The culture medium was then removed, and the cells were incubated for 1 h in incubation buffer containing 15 mM HEPES (pH 7.6), 135 mM NaCl, 1.8 mM CaCl_2_, and 0.8 mM MgCl_2_. The cells were then exposed for 15 min to incubation buffer containing freshly prepared hydrogen peroxide at the following concentrations: 0.01, 0.1, or 1 mM H_2_O_2_. At the end of the incubation period, the cells were washed twice with incubation buffer and subsequently cultured in complete growth medium at 37 °C in a humidified atmosphere of 5% CO_2_ for the desired period.

### Cell viability

Cell viability was determined by trypan blue exclusion using a Neubauer hemacytometer. For this, 50 µL of trypsin solution (0.05% trypsin and 0.53 mM EDTA, pH 8.0) was added to each well, and the cells were incubated at 37 °C for 1 min. The cells were then resuspended in culture medium, and 100 µL of the cell suspension was mixed with 100 µL of trypan blue in an Eppendorf tube. The cells were subsequently counted using a hemacytometer, and cell viability was determined by the trypan blue exclusion method. All experiments were done in triplicate, including the untreated controls.

### Statistical analysis

Two independent experiments were performed, each including three technical replicates per condition. Technical replicates were not considered independent observations. Standard deviation (SD) is shown only as a descriptive measure of technical variability within the experiment presented. Given the limited number of independent experiments (n = 2), no inferential statistical analysis was performed.

## Results

In initial experiments, HUVECs were cultured for 24 h in medium conditioned with 0.1, 1.0 and 10 Eq of cigarette smoke, and the effect on cell viability and the content of glutathione was monitored. Four conditions were tested: i) Cells containing glutathione (2.5–4 mM) and cultured in medium lacking vitamin C; ii) cells containing glutathione and preloaded with vitamin C (1.5–2 mM intracellular AA); iii) cells lacking glutathione (< 50 µM) after treatment with 1 mM BSO and 1 mM DEM and cultured in medium lacking vitamin C; and iv) cells lacking glutathione and preloaded with vitamin C. HUVECs exposed to cigarette smoke-conditioned medium showed changes in cell viability and glutathione content that were concentration and time-dependent, with survival showing a marked dependency on the presence of intracellular glutathione and no evidence for an effect of intracellular vitamin C (Figure 1). Glutathione-containing cells exposed to medium conditioned with 10 Eq of cigarette smoke showed a progressive time-dependent decrease in cell viability, with approximately 35% of cells surviving a 24-h treatment (Figure 1A), and similar results (30% survival) were obtained in cells containing glutathione and supplemented with vitamin C (Figure 1B). On the other hand, there was less than 3% survival in cells lacking glutathione that were unsupplemented (Figure 1C) or supplemented with vitamin C (Figure 1D). Confirming these results, although greater than 95% of glutathione-containing cells (with or without vitamin C) survived a 24-h exposure to medium conditioned with 0.1 and 1 Eq of cigarette smoke (Figures 1A,B), cells lacking glutathione showed decreased survival in the range of 70%–80% (Figures 1C,D). Cells containing glutathione (with or without vitamin C) showed a biphasic response to cigarette smoke-conditioned medium, with an initial phase that lasted 8 h characterized by a decrease in cell survival, followed by a secondary phase for the next 12 h in which that tendency was reverted with a clear recovery of cell survival (Figures 1A,B). In contrast, cells lacking glutathione (with or without vitamin C) showed a continuous decrease in cell survival for the 24 h of the experiment and lacked the reversion step (Figures 1C,D). Overall, cell viability at 24 h depended primarily on the presence of intracellular glutathione, independently of vitamin C supplementation.

**FIGURE 1:**
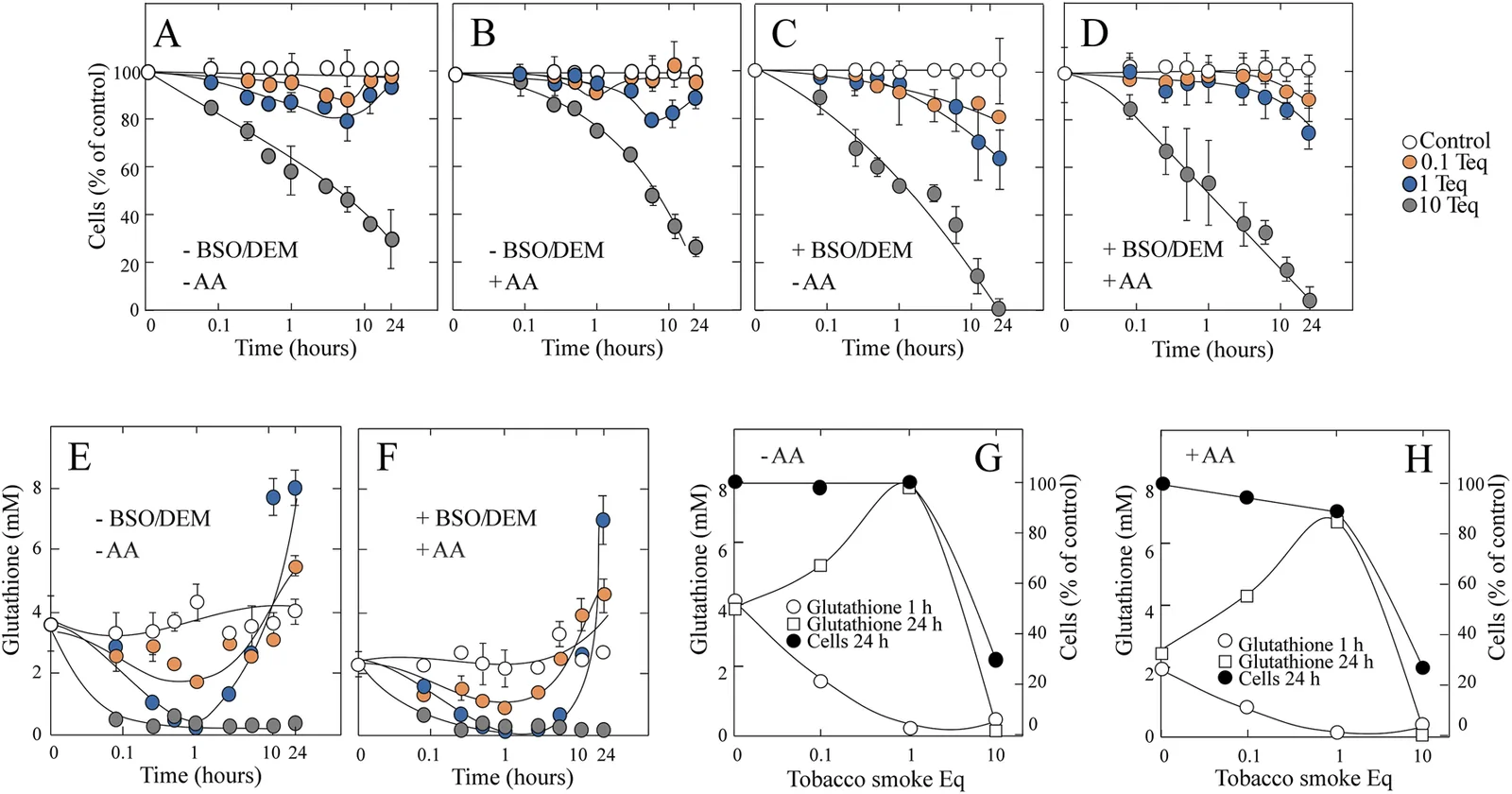
Cell viability and glutathione content in HUVECs exposed to cigarette smoke-conditioned medium for 24 h. HUVECs were incubated for 24 h with medium conditioned with 0 (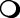), 0.1 (●), 1 (●) and 10 Eq of cigarette smoke (●), and cell viability and glutathione content were measured at different times. **(A–D)** Cell viability was measured using trypan blue exclusion in the following conditions: Control cells with normal levels of glutathione **(A)**, control cells plus AA **(B)**, BSO/DEM-treated cells **(C)** and BSO/DEM-treated cells plus AA **(D)**. **(E,F)** Glutathione content in control cells with no AA **(E)** and with AA **(F)**. **(G,H)** Comparison between glutathione content at 1 (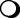) and 24 h (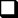), and cell viability at 24 h (●), in control cells **(G)** or control cells plus AA **(H)** and exposed to increasing Eq of cigarette smoke. AA indicates ascorbic acid supplementation; BSO/DEM indicates treatment with L-buthionine-(S,R)-sulfoximine and diethyl maleate for glutathione depletion. Non-zero time points are displayed using logarithmic spacing; 0 h represents the baseline time point. Data represent the mean ± standard deviation (SD) of three technical replicates from the experiment shown. Two independent experiments were performed and showed comparable response patterns. The SD reflects technical variability within the experiment and does not represent variability between independent experiments.

We next analyzed whether HUVECs exposed to cigarette smoke-conditioned medium affected their glutathione content and if vitamin C plays a role in the antioxidant defense. Control cells showed relatively stable levels of glutathione of around 3.5–4.0 mM (Figures 1E,F), while cells cultured in cigarette smoke-conditioned media showed a concentration and time-dependent biphasic change in glutathione (Figure 1E). There was a rapid decrease in glutathione that reached a minimal level within the first hour of culture, followed by a recovery period with an overshoot at 24 h (Figure 1E). The glutathione content at 1 h was inversely related to the Eq of cigarette smoke used but was unrelated to the extent of cell survival (Figure 1G). Although the secondary glutathione recovery phase was more complex, higher glutathione concentrations at 24 h were associated with higher cell viability (Figure 1G). Thus, cells exposed to medium conditioned with 0.1 and 1 Eq of cigarette smoke could recover their glutathione content and survived the oxidative challenge, while those in which glutathione levels remained low (10 Eq of cigarette smoke) showed the lowest viability (Figure 1G). Similar results were obtained when using cells containing glutathione and supplemented with vitamin C and cultured in media conditioned with increasing Eq of cigarette smoke: i) a concentration- and time-dependent biphasic change in their glutathione content similar to that of cells lacking vitamin C (Figure 1F); ii) the glutathione content at 1 h was inversely related to the Eq of cigarette smoke used and unrelated to cell survival (Figure 1H); iii) for the secondary phase, only cells exposed to medium conditioned with 1 or with 0.1 Eq of cigarette smoke recovered their glutathione content and survived the oxidative challenge, while cells cultured in medium conditioned with 10 Eq of cigarette smoke lost their glutathione very rapidly, were unable to recover it during the first phase of the experiment and failed to survive (Figure 1H). Overall, our results indicate that the ability of HUVECs to survive oxidative challenge when exposed to soluble cigarette smoke toxicants is directly related to the capacity to recover their glutathione content, a process that appears to be independent of vitamin C.

The effects of prolonged exposure to soluble cigarette smoke toxicants on HUVEC survival were examined by culturing cells for up to 7 days in medium conditioned with 1 Eq of cigarette smoke. Cell viability was primarily dependent on intracellular glutathione content. Cells containing glutathione, with or without vitamin C supplementation, survived normally during continuous exposure to cigarette smoke, displaying viability comparable to control cells (Figures 2A,B). In contrast, glutathione-depleted control cells remained viable but failed to increase in viable cell number (Figure 2C). When glutathione-depleted cells were exposed to cigarette smoke-conditioned medium, a pronounced time-dependent decrease in cell number was observed, regardless of vitamin C supplementation, and no viable cells were detected on days 6 and 7 (Figures 2C,D). Vitamin C supplementation exerted only a modest and transient protective effect during the first 24–48 h in glutathione-depleted cells (Figure 2D), which was lost at later time points. Together, these results indicate that intracellular glutathione is the principal determinant of endothelial cell survival during prolonged exposure to soluble CSM (Figures 2A–D).

**FIGURE 2:**
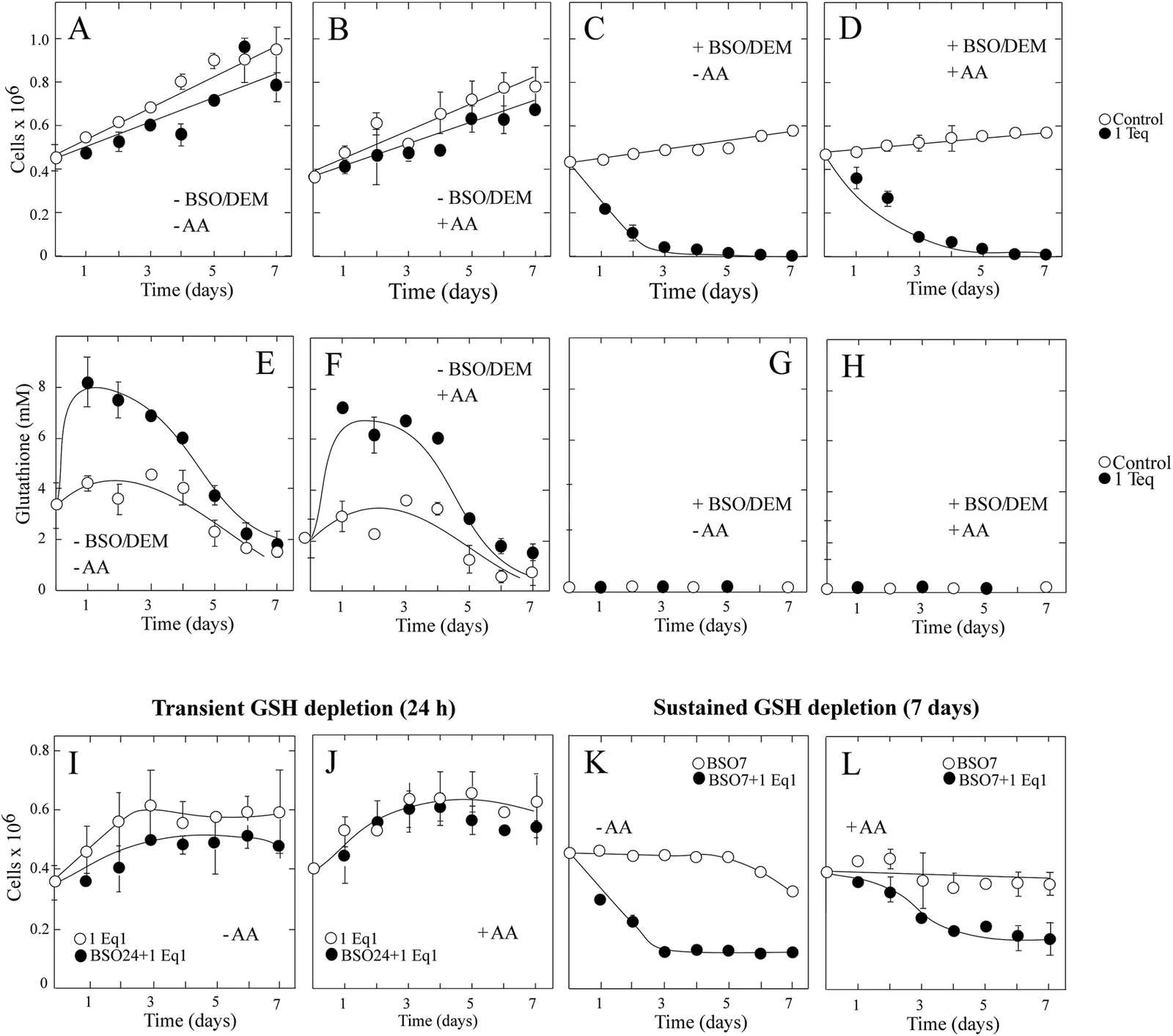
Effects of continuous and transient exposure to cigarette smoke-conditioned medium on cell viability and intracellular glutathione content in HUVECs. **(A–D)** Time course of viable cell number during continuous exposure to cigarette smoke-conditioned medium. HUVECs were cultured for 7 days in untreated culture medium (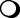) or in medium conditioned with 1 Eq of cigarette smoke (●). Medium was replaced every 24 h with the following conditions: Control cells cultured without AA **(A)** control cells loaded with AA **(B)** BSO/DEM-treated cells cultured without AA **(C)** and BSO/DEM-treated cells loaded with AA **(D)**. **(E–H)** Time course of intracellular glutathione content during continuous exposure to untreated culture medium (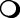) or medium conditioned with 1 Eq of cigarette smoke (●). E-H, Cells were treated with the following conditions: Control cells cultured without AA **(E)** control cells loaded with AA **(F)** BSO/DEM-treated cells cultured without AA **(G)** and BSO/DEM-treated cells loaded with AA **(H)**. **(I,J)** Time course of viable cell number following transient exposure to cigarette smoke-conditioned medium. Cells exposed to medium conditioned with 1 Eq of cigarette smoke for 24 h and subsequently cultured for an additional 6 days in cigarette smoke-free medium are represented by an open circle (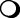). Cells pretreated with BSO for 24 h (BSO24) before exposure to medium conditioned with 1 Eq of cigarette smoke for 24 h, followed by an additional 6 days in cigarette smoke-free medium are represented by a filled circle (●). Cells were cultured without **(I)** or loaded with AA **(J)**. **(K,L)** Time course of viable cell number in glutathione-depleted cells maintained in BSO-containing medium for 7 days (BSO7). Open circles (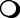) represent cells maintained with BSO without exposure to cigarette smoke, whereas filled circles (●) represent cells maintained with BSO and exposed to medium conditioned with 1 Eq of cigarette smoke during the first 24 h. Cells were cultured without AA **(K)** or loaded with AA **(L)**. AA indicates ascorbic acid supplementation; BSO/DEM indicates treatment with L-buthionine-(S,R)-sulfoximine and diethyl maleate for glutathione depletion. Data represent the mean ± standard deviation (SD) of three technical replicates from the experiment shown. Two independent experiments were performed and showed comparable response patterns. The SD reflects technical variability within the experiment and does not represent variability between independent experiments.

Control cells showed glutathione levels of around 3.5–4.0 mM during the first 4 days, with a progressive decline afterwards that was time-dependent and reached a glutathione concentration of 1.5 mM at day seven (Figure 2E). Exposure to medium conditioned with 1 Eq of cigarette smoke was accompanied by an initial increase in the glutathione content, with a maximum value of 8 mM at day one, followed by a time-dependent decrease and a glutathione concentration of 2 mM at day seven (Figure 2E). A similar biphasic behaviour was observed in cells containing glutathione and vitamin C and treated with medium conditioned with 1 Eq of cigarette smoke; a doubling of intracellular glutathione (about 6 mM) during the first 4 days of treatment, followed by a continuous decline to reach 1.5 mM at day seven, compared with 1 mM in control cells (Figure 2F). Cells were treated with BSO and cultured. During the first 5 days of the experiment, no glutathione was detected in BSO-treated cells (Figures 2G,H). In cells exposed to 1 Eq of cigarette smoke, no viable cells were observed on days 6 and 7 (Figures 2C,D). These results confirmed the lack of relevance of vitamin C in protecting the endothelial cells from the deleterious effect of exposure to soluble CSM, and the central role of glutathione in cell survival (Figures 2E–H).

In the previous experiments, the endothelial cells were continuously exposed to soluble CSM. We asked whether cells treated for 24 h with cigarette smoke-conditioned medium and then cultured in normal medium would respond differently to glutathione and vitamin C. These experiments confirmed the central role of glutathione in cell survival (Figures 2I–L). BSO-treated cells showed a lower survival level than control cells during the first 5 days (Figure 2I). In vitamin C-supplemented cells, the difference between BSO-treated and control cells was less pronounced (Figure 2J).

Cells lacking both glutathione and vitamin C showed a continuous decrease in survival from day one to three, reaching 30% survival at day 3 that was maintained through day seven (Figure 2K). In comparison, cells supplemented with vitamin C alone showed a survival level similar to control cells during the first 2 days, followed by a major decrease in survival at day three that decreased to 40% from day four through seven (Figure 2L), pointing to a transient effect of vitamin C on cell survival in the absence of glutathione. Overall, these results confirmed the central role of glutathione in determining the survival of endothelial cells exposed to soluble smoke toxicants.

To investigate whether a state of oxidative stress induced by soluble CSM affected the general antioxidant defense capacity of endothelial cells exposed to oxidants that are normally present as a result of cellular metabolism, we treated the endothelial cells with cigarette smoke-conditioned media, followed by treatment with graded concentrations of hydrogen peroxide and studied the effect on cell viability, the content of glutathione and the role of vitamin C. Cells were incubated for 1 h with culture medium conditioned with 1 Eq of cigarette smoke and subsequently exposed for 15 min to medium containing 0.01, 0.1, or 1 mM hydrogen peroxide (H_2_O_2_). After H_2_O_2_ exposure, the cells were washed and maintained in H_2_O_2_-free culture medium for an additional 24 h, during which cell viability and intracellular glutathione levels were monitored. These experimental conditions were selected because a 1-h exposure to medium conditioned with 1 Eq of cigarette smoke markedly reduced the intracellular soluble glutathione pool. Similarly, a 15-min exposure to H_2_O_2_ reduced measurable intracellular glutathione to the lowest levels detected under our experimental conditions.

For cells containing glutathione but lacking vitamin C, there was a dose and time-dependent decrease in cell viability as a result of the treatment with cigarette smoke-conditioned medium and hydrogen peroxide (Figures 3A–D). About 75, 35% and 10% of the endothelial cells survived treatment with 0.01, 0.1 and 1.0 mM hydrogen peroxide at 24 h respectively (Figure 3A), and vitamin C supplementation failed to affect cell viability (Figure 3B). These cells showed survival rates similar to cells containing glutathione but lacking vitamin C. As expected from our previous results, cells lacking glutathione showed a markedly augmented sensitivity to treatment, which was independent of the absence or the presence of vitamin C (Figures 3C,D). Thus, less than 20% of the cells survived treatment with cigarette smoke-conditioned medium plus 0.01 mM hydrogen peroxide for 24 h, and no cells survived treatment with 1 mM hydrogen peroxide (Figures 3C,D). In parallel studies, we measured the glutathione content of cells subjected to the different treatments to assess whether their viability was related to their capacity to recover their glutathione content and the role of glutathione and vitamin C in the overall process. Cells treated with cigarette smoke-conditioned medium plus hydrogen peroxide showed higher levels of glutathione at 24 h (Figures 3E,F). Thus, for cells containing glutathione at the start of the experiment (1.5–1.8 mM), there was an initial lag period that lasted about 10 min, followed by a secondary period in which there was a major increase in intracellular glutathione, with the result that at 24 h the cells contained glutathione levels in the range of 6–12 mM (Figures 3E,F). Interestingly, there was no clear relationship between glutathione content and cell survival at 24 h. Vitamin C supplementation failed to enhance glutathione recovery or confer an evident survival advantage in cells treated with cigarette smoke-conditioned medium plus hydrogen peroxide. Also, cells depleted of glutathione before being subjected to oxidative challenge with cigarette smoke-conditioned medium plus hydrogen peroxide were not able to recover their glutathione content (Figures 3G,H), and their capacity to recover their glutathione content was limited, with a 24-h value almost one order of magnitude lower (0.8–1.2 mM) to cells containing glutathione (Figures 3G,H). Moreover, vitamin C supplementation failed to increase the rate of glutathione recovery (Figure 3H).

**FIGURE 3:**
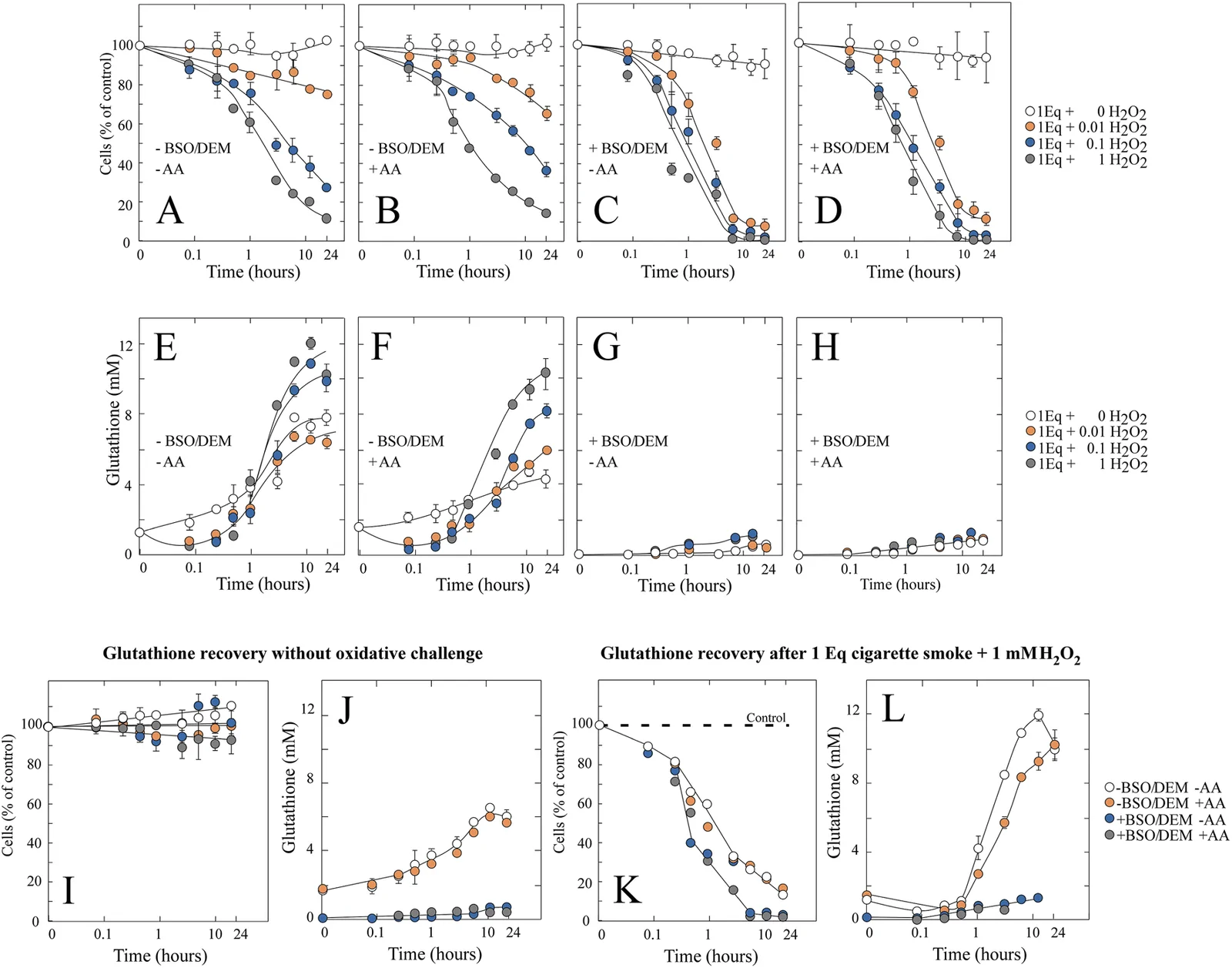
Cell viability and glutathione content in endothelial cells exposed to cigarette smoke-conditioned medium and hydrogen peroxide. **(A-D)** Effect on cell viability. HUVECs were incubated for 1 h in medium conditioned with 1 Eq of cigarette smoke and then 15 min with medium containing 0 (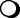), 0.01 (●), 0.1 (●) and 1 mM hydrogen peroxide (●), washed and cultured in hydrogen peroxide-free medium for 24 h, and cell viability was measured at different times. Cells contained glutathione alone **(A)** or glutathione plus vitamin C **(B)** neither glutathione nor vitamin C **(C)** or vitamin C alone **(D)**. **(E–H)** Effect on glutathione content in endothelial cells exposed to cigarette smoke-conditioned medium and hydrogen peroxide. HUVECs were incubated for 1 h in medium conditioned with 1 Eq of cigarette smoke and then 15 min with medium containing 0 (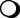), 0.01 (●), 0.1 (●) and 1 mM hydrogen peroxide (●), washed and cultured in hydrogen peroxide-free medium for 24 h, and intracellular glutathione content was measured at different times. Cells contained glutathione alone **(E)** or glutathione plus vitamin C **(F)** neither glutathione nor vitamin C **(G)** or vitamin C alone **(H)**. **(I,J)** Glutathione recovery without oxidative challenge. Cells were incubated for 24 h, and cell viability **(I)** and glutathione content **(J)** were measured at different times. **(K,L)** Glutathione recovery capacity after 1 Eq of cigarette smoke plus 1 mM hydrogen peroxide. Cell viability **(K)** and glutathione content **(L)** were measured at different times. Cells contained glutathione alone (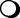), or glutathione plus vitamin C (●), neither glutathione nor vitamin C (●), or vitamin C alone (●). AA indicates ascorbic acid supplementation; BSO/DEM indicates treatment with L-buthionine-(S,R)-sulfoximine and diethyl maleate for glutathione depletion. Non-zero time points are displayed using logarithmic spacing; 0 h represents the baseline time point. Data represent the mean ± standard deviation (SD) of three technical replicates from the experiment shown. Two independent experiments were performed and showed comparable response patterns. The SD reflects technical variability within the experiment and does not represent variability between independent experiments.

We tested whether the low capability of cells lacking glutathione from the start to recover and increase their glutathione content was due to the treatment with conditioned medium or was critically dependent on the initial glutathione content. For this, we obtained cells containing glutathione alone, glutathione plus vitamin C, lacking both glutathione and vitamin C, and containing vitamin C alone, and measured cell viability and glutathione during a 24-h period. There were only minor differences in cell survival at 24 h, independently of the glutathione status of the cells (Figure 3I). These experiments also showed that cells containing glutathione (1.8 mM) showed a continuous increase in glutathione during the 24-h assay (3-fold increase), reaching a final glutathione concentration of 6 mM (Figure 3J), while cells lacking glutathione showed a very limited glutathione recovery capacity, with levels at 24 h below 0.8 mM (Figure 3J). We next used cells treated with medium conditioned with 1 Eq of cigarette smoke followed by exposure to 1 mM hydrogen peroxide, a condition that we already know will kill most cells, and measured cell survival and glutathione for 24 h. As expected, cell viability was very low in glutathione-containing cells (< 15%), and no glutathione-lacking cells survived the first 12 h (Figure 3K). Interestingly, the few surviving cells from the treated glutathione-containing cells showed a marked capacity to recover their intracellular soluble glutathione, which reached values near 12 mM (an 8-fold increase) at 24 h (Figure 3L). In contrast, the cells lacking glutathione showed a very limited glutathione recovery capacity, with levels at 24 h below 1.2 mM (Figure 3L). Overall, these results indicate that the capacity of the endothelial cells to survive the oxidative challenge depends on two factors that may act in concert: i) the content of glutathione at the start of the oxidative challenge, and ii) the severity of the oxidative challenge.

We have previously shown that HUVECs express the sodium-dependent vitamin C transporter 2 (SVCT2), encoded by the SLC23A2 gene, and efficiently transport the reduced form of vitamin C, ascorbic acid (Montecinos et al., 2007). We therefore assessed whether treatment of endothelial cells with cigarette smoke-conditioned medium affected their capacity to transport AA. Our results from time-course uptake experiments revealed that ascorbic acid transport was not affected by treating the endothelial cells for 24 h with medium conditioned with 1 Eq of cigarette smoke and was also independent of the cellular content of glutathione or vitamin C, alone or in combination (Figures 4A–D). Similar results were obtained when measuring the transport of AA in cells containing glutathione without or with vitamin C cultured for 7 days with medium (changed daily) conditioned with 1 Eq of cigarette smoke, with similar AA transport rates for control and treated cells (Figures 4E,F). However, cells lacking vitamin C transported AA at a rate that was approximately 2.5-fold faster than cells supplemented with vitamin C (Figures 4E,F). We next tested the capacity of the endothelial cells to transport the oxidized form of vitamin C, dehydroascorbic acid, which is transported by members of the glucose transporter family, or GLUTs (Mardones, 2025; Rivas et al., 2008). These experiments revealed that the time-dependent uptake curve is biphasic (Figures 4G–J), with a short initial period of rapid uptake that corresponds to the actual transport phase, followed by a secondary, slower phase that represents the intracellular accumulation of the transported substrate (Munoz-Montesino et al., 2021; Vera et al., 1995). These results revealed that, neither the initial DHA transport rate nor the accumulation step, was affected by treating the endothelial cells for 24 h with medium conditioned with 1 Eq of cigarette smoke, or by the cellular content of glutathione or vitamin C, alone or in combination (Figures 4G–J). On the other hand, although uptake experiments performed after 7 days of culture in cigarette smoke-conditioned medium revealed no effect on the transport rate, we observed an approximately two-fold increase in the capacity of the treated cells to accumulate vitamin C compared with the respective control cells (Figures 4K,L). We conclude that a short exposure of endothelial cells to soluble CSM failed to affect the capacity of the endothelial cells to transport vitamin C, in its reduced or oxidized form, which is consistent with our finding that glutathione, and not vitamin C, is the soluble antioxidant that determines the resistance of endothelial cells to the deleterious effect of cigarette smoke.

**FIGURE 4:**
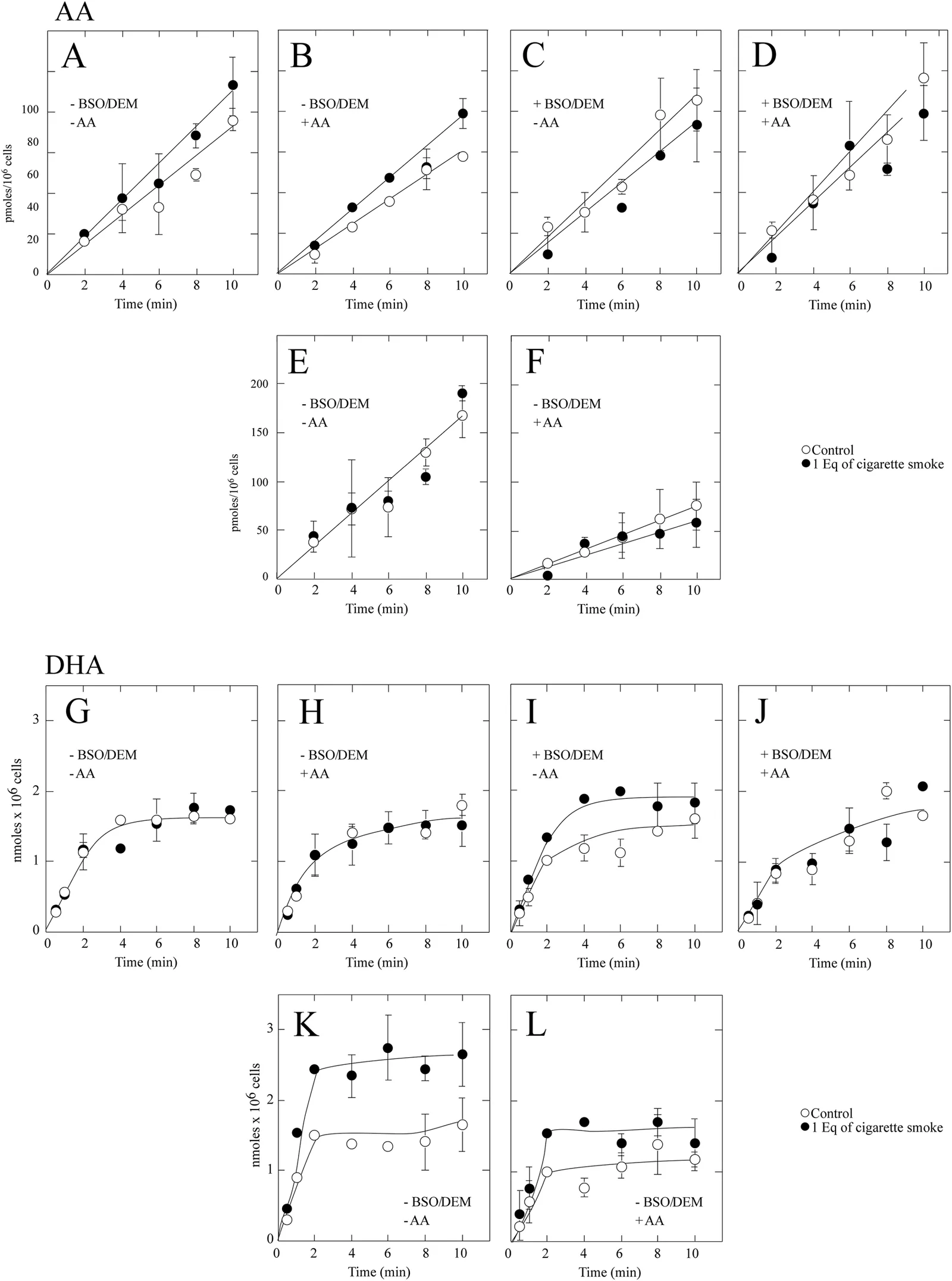
Transport and accumulation of vitamin C in endothelial cells treated with cigarette smoke-conditioned medium. **(A–F)** Ascorbic acid transport. HUVECs were incubated for 24 h **(A–D)** or 7 days **(E,F)** with (●) or without (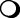) cigarette smoke-conditioned medium (1 Eq) before measuring ascorbic acid transport. Cells contained glutathione alone **(A,E)**, glutathione plus vitamin C **(B,F)**, neither glutathione nor vitamin C **(C)**, or vitamin C alone **(D)**. (**G–L**) Dehydroascorbic acid uptake. HUVECs were incubated for 24 h **(G–J)** or 7 days **(K,L)** with (●) or without (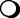) cigarette smoke-conditioned medium (1 Eq) before measuring dehydroascorbic acid uptake. Cells contained glutathione alone **(G,K)** glutathione plus vitamin C **(H,L)** neither glutathione nor vitamin C **(I)** or vitamin C alone **(J)**. Transport data were obtained from the initial phase of rapid uptake in the time-course uptake curve (≤ 2 min), while accumulation was estimated from the slower secondary phase (≥ 2 min). AA indicates ascorbic acid supplementation; BSO/DEM indicates treatment with L-buthionine-(S,R)-sulfoximine and diethyl maleate for glutathione depletion. Data represent the mean ± standard deviation (SD) of three technical replicates from the experiment shown. Two independent experiments were performed and showed comparable response patterns. The SD reflects technical variability within the experiment and does not represent variability between independent experiments.

## Discussion

Our present results provide new information on the functional relationship between glutathione and vitamin C in endothelial cells exposed to oxidative challenge induced by soluble cigarette smoke toxicants. Functional interactions between glutathione and vitamin C have been demonstrated *in vivo* in experimental models of glutathione or vitamin C deficiency and *in vitro* in endothelial cells subjected to oxidative challenge (Mardones et al., 2012; Montecinos et al., 2007; Strand et al., 2025). These studies indicate that glutathione and vitamin C can cooperate in antioxidant defense. However, our present results indicate that, under exposure to soluble cigarette smoke toxicants, intracellular glutathione plays a central role in endothelial cell survival and that vitamin C supplementation cannot fully compensate for glutathione depletion.

In this context, our results can be explained in terms of the changes in intracellular glutathione in cells exposed to soluble cigarette smoke toxicants. Treatment of endothelial cells with cigarette smoke-conditioned medium resulted in a dose- and time-dependent decrease in cell viability that was preceded by a rapid decrease in measurable intracellular glutathione.

The initial decrease in glutathione was related to the amount of cigarette smoke Eq but was not directly related to the capacity of the cells to survive the oxidative challenge. Instead, cell survival was associated with the capacity of the endothelial cells to recover their glutathione content during the following 24 h, reaching concentrations three-to fourfold higher than those present in untreated control cells.

Because the enzymatic recycling assay used in this study quantified soluble GSH plus GSSG in the supernatant obtained after protein precipitation, the observed decrease cannot be attributed exclusively to oxidation of GSH to GSSG. It may also reflect glutathione export and/or redistribution into protein-bound or other non-measured glutathione pools. Therefore, our present experiments do not distinguish between the different mechanisms that may contribute to the rapid decrease in measurable intracellular glutathione.

The importance of glutathione recovery was also supported by the experiments in which glutathione synthesis was inhibited with BSO. Cells lacking glutathione at the onset of the experiment showed a limited capacity to recover their glutathione content and were more susceptible to cigarette smoke-conditioned medium. During prolonged exposure, cells containing glutathione remained viable with or without vitamin C supplementation, whereas glutathione-depleted cells showed a progressive decrease in viability.

Vitamin C supplementation produced only a modest and transient protective effect during the first 24–48 h and failed to prevent the subsequent decrease in cell viability. Overall, these results indicate that the glutathione content at the onset of the oxidative challenge and the capacity of the cells to recover their intracellular glutathione are important factors in determining endothelial cell survival during exposure to soluble cigarette smoke toxicants.

The central role of glutathione may be related, at least in part, to the chemical characteristics of cigarette smoke toxicants. Previous studies have shown that components of cigarette smoke can react with glutathione and generate stable glutathione adducts, thereby decreasing the available intracellular GSH pool (van der Toorn et al., 2007). This mechanism could contribute to the rapid decrease in glutathione observed in our experiments, although we did not directly determine the products generated from intracellular glutathione.

The relevance of the initial glutathione content was further supported by the experiments in which endothelial cells exposed to cigarette smoke-conditioned medium were subsequently challenged with hydrogen peroxide. Under these conditions, cell viability was markedly decreased, particularly in cells lacking glutathione, and vitamin C supplementation failed to enhance glutathione recovery or provide an evident advantage in cell survival. These results indicate that the capacity of endothelial cells to survive the oxidative challenge depends on both the glutathione content at the start of the experiment and the severity of the oxidative challenge.

An additional finding of our study was that exposure to cigarette smoke-conditioned medium failed to decrease the capacity of endothelial cells to transport vitamin C. Treatment with cigarette smoke-conditioned medium did not affect the transport of either ascorbic acid or dehydroascorbic acid, independently of the intracellular content of glutathione or vitamin C.

Similarly, prolonged exposure for 7 days failed to decrease vitamin C transport, although an increased accumulation of vitamin C was observed in treated cells. Therefore, the limited protective effect of vitamin C observed under our experimental conditions does not appear to result from a decreased capacity of endothelial cells to acquire vitamin C from the extracellular medium. It is important to note, however, that these experiments specifically examined vitamin C transport and accumulation and do not allow us to conclude that all aspects of intracellular vitamin C metabolism were unaffected by cigarette smoke exposure.

Our present findings are consistent with previous studies examining the relationship between vitamin C, glutathione, and cigarette smoke exposure. Diverse studies have analyzed changes in vitamin C levels in smokers and individuals exposed to cigarette smoke without reaching a clear conclusion regarding the capacity of vitamin C supplementation to counteract the negative effects associated with smoking (Ashor et al., 2014; Astori et al., 2022; Bruno et al., 2005; Dietrich et al., 2003; Karademirci et al., 2018; Lykkesfeldt and Tveden-Nyborg, 2019; Panda et al., 2000; Preston et al., 2003; Strauss, 2001; Wilson et al., 2011).

Additional studies have revealed that glutathione appears to play an important role in antioxidant defense during cigarette smoke exposure *in vitro* as well as *in vivo*, although the contribution of vitamin C was not always examined simultaneously (Baglole et al., 2008; Conklin et al., 2009; Dalle-Donne et al., 2020; Horiyama et al., 2016; Luchese et al., 2009; Muscat et al., 2004; Shin et al., 2020; van der Toorn et al., 2007).

Our present results extend these observations by showing that, in primary endothelial cells exposed to soluble cigarette smoke toxicants, glutathione availability and the capacity to recover intracellular glutathione are closely related to cell survival, whereas vitamin C supplementation cannot fully compensate for glutathione depletion.

The present results were obtained using an *in vitro* experimental model with primary HUVECs exposed to the water-soluble fraction of cigarette smoke and therefore do not reproduce all the effects associated with cigarette smoke exposure *in vivo*. Most importantly, our results consistently indicate that intracellular glutathione availability is central to the capacity of endothelial cells to survive oxidative challenge induced by soluble cigarette smoke toxicants in a manner that appears to be independent of the vitamin C status.

These findings suggest further investigation are required to determine the mechanisms involved in glutathione recovery and to establish whether maintaining or restoring intracellular glutathione levels can increase endothelial cell resistance to cigarette smoke-associated oxidative stress challenge.

## Data Availability

The original contributions presented in the study are included in the article/supplementary material, further inquiries can be directed to the corresponding authors.

## In memoriam

Dedicated to the memory of our mentor Juan Carlos Vera, whose contribution to this work was invaluable.
